# Breast Cancer in a 53-year-old Obese Male

**DOI:** 10.7759/cureus.6194

**Published:** 2019-11-19

**Authors:** Joel Robinette, Rob Olexo

**Affiliations:** 1 Otolaryngology / Surgery, West Virginia School of Osteopathic Medicine, Lewisburg, USA; 2 Family Medicine, West Virginia School of Osteopathic Medicine, Lewisburg, USA

**Keywords:** breast cancer, estrogenic overproduction, male breast cancer, risk factor

## Abstract

Male breast cancer (MBC) represents <1% of all breast cancers and little is known about its true etiology. The known risk factors associated with MBC are age, Klinefelter syndrome, BRCA2 mutation, high estrogen levels, gynecomastia, and cirrhosis of any cause. Obesity has been documented as a risk factor to MBC in some studies even though it is not officially recognized as a risk factor. Herein, we present a 53-year-old obese male with estrogen receptor-positive (ER+) breast cancer. Although this patient has a strong family history of ovarian cancer, obesity could have been an additive predisposing factor. As more cases of MBC in obese patients are explored, we might be able to gain a better understanding of its true etiology and mechanism.

## Introduction

Male breast cancer (MBC) represents <1% of all breast cancers and little is known about its true etiology. Although rare, the incidence of MBC is rising. A large population-based study reported the incidence of MBC has increased from 0.086 to 1.08 per 100,000 from 1973 to 1998 [1]. Obesity has also become a larger issue as the incidence of MBC has increased [2]. This increased male obesity and MBC seem to mirror each other [2].

We present the case of a 53-year-old morbidly obese male diagnosed with estrogen receptor-positive (ER+) stage IIIa ductal carcinoma in situ (DCIS) breast cancer and is currently being treated. While there are many known and recognized risk factors for MBC, obesity is not officially recognized. Recent studies show evidence of obesity as a risk factor [3]. Although this patient has a strong family history of ovarian cancer, obesity could have been an additive predisposing factor. 

## Case presentation

A 53-year-old male presented to the office complaining of a lump in his left breast. The patient noticed his left nipple was inverted about a month prior that was not present in his right breast. Lump measured 17-mm firm and was mildly tender, and the patient stated the lump had grown in the past month. The patient weighed 241 lbs and had a BMI of 37 kg/m^2^ and also a history of gastric bypass surgery for obesity. The patient also had a strong family history of ovarian cancer.

The radiological report included transverse and longitudinal scans throughout the left breast and found roughly a 17-mm irregular area of decreased echogenicity in the 12 o’clock region adjacent to the nipple and flow within. Biopsy demonstrated invasive ductal carcinoma, Nottingham grade 2, pathologic T1c, ER+, progesterone receptor-negative, and HER-2/neu negative by immunohistochemistry. Subsequent left simple mastectomy with axillary lymph node dissection was performed, and six out of 19 lymph nodes were positive for metastatic breast cancer demonstrating N2a. The patient underwent dose-dense adjuvant chemotherapy with Adriamycin/Cytoxan, following which he was recommended adjuvant hormonal therapy for 10 years.

## Discussion

As mentioned above, male obesity and MBC are related [2]. In an obese male, the excess adipose tissue provides an adequate environment for testosterone to be converted to estrogen via increased aromatase. Obese males produce twice as much estrogen than men with an average BMI [4]. Our patient had a BMI of 37 kg/m^2^ after his history of gastric bypass surgery, which corresponds to a morbidly obese male with potentially chronic hyperestrogenism. Although gynecomastia may be related to obesity, its evidence as a risk factor for MBC is also unclear; 40% of breast cancer patients demonstrated microscopic evidence of gynecomastia [5-6]. Other autopsy studies reported gynecomastia in 50% of MBC patients [7]. Our patient showed significant gynecomastia. Weight gain in perimenopausal women has been demonstrated as a risk factor for breast cancer and correlates with an adverse ER+ risk factor [8]. 

The known risk factors associated with MBC are age, Kleinfelter syndrome, BRCA2 mutation, high estrogen levels, gynecomastia, and cirrhosis from chronic alcohol use [2]. Obesity has been documented as a risk factor to MBC in some studies even though it is not officially recognized as a risk factor [2]. Our patient's chronic obesity, along with his family history of ovarian cancer, may have a strong correlation with his development of ER+ breast cancer. The Male Breast Cancer Pooling demonstrated that obesity was a positive risk factor for MBC (30% increase) from 10 cohort studies observing over 2,400 patients [3].

In this case report, we have presented an obese male with ER+ breast cancer. Although this patient has a strong family history of ovarian cancer, obesity could have been an additive predisposing risk factor. White et al. noted that reasons for the rise in MBC can only be speculated but recognized that obesity has also increased and may be linked to breast cancer [9].

## Conclusions

Male breast cancer is rare, and little is known about its true etiology. Obesity has been documented as a risk factor to MBC in some studies even though it is not officially recognized as a risk factor. Although this patient has a strong family history of ovarian cancer, obesity could have been an additive predisposing factor. As more cases of male breast cancer in obese patients are explored, we might be able to gain a better understanding of its true etiology and mechanism.

## References

[1] Giordano S, Cohen D, Buzdar A, Perkins G, Hortobagyi G (2004). Breast carcinoma in men: a population-based study. Cancer.

[2] Humphries MP, Jordan VC, Speirs V (2015;13). Obesity and male breast cancer: provocative parallels?. BMC Med.

[3] Brinton LA, Cook MB, McCormack V (2014). Anthropometric and hormonal risk factors for male breast cancer: male breast cancer pooling project results. J Natl Cancer Inst.

[4] Schneider G, Kirschner MA, Berkowitz R, Ertel NH (1979). Increased estrogen production in obese men. J Clin Endocrinol Metab.

[5] Weiss JR, Moysich KB, Swede H (2005). Epidemiology of male breast cancer. Cancer Epidemiol Biomark Prev.

[6] Heller KS, Rosen PP, Schottenfeld D, Ashikari R, Kinne DW (1978). Male breast cancer: a clinicopathologic study of 97 cases. Ann Surg.

[7] Andersen JA, Gram JB (1982). Male breast at autopsy. Acta Pathol Microbiol Immunol Scand Suppl.

[8] Scholz C, Andergassen U, Hepp P Obesity as an independent risk factor for decreased survival in node-positive high-risk breast cancer. Breast Cancer Res Treat.

[9] White J, Kearins O, Dodwell D, Horgan K, Hanby AM, Speirs V (2011). Male breast carcinoma: increased awareness needed. Breast Cancer Res.

